# CAI4CAI: The Rise of Contextual Artificial Intelligence in Computer-Assisted Interventions: This article overviews ideas as to how to incorporate the range of prior knowledge and instantaneous sensory information from experts, sensors and actuators for use in computer-assisted interventions, as well as learning how to develop a representation of the surgery or intervention among a mixed human-AI team of actors. In addition, the design of interventional systems and associated cognitive shared control schemes for online uncertainty awareness when making decisions in the OR or the IR suite is discussed, and it is noted how this is critical for producing precise and reliable interventions.

**DOI:** 10.1109/JPROC.2019.2946993

**Published:** 2019-10-23

**Authors:** Tom Vercauteren, Mathias Unberath, Nicolas Padoy, Nassir Navab

**Affiliations:** 1 School of Biomedical Engineering & Imaging SciencesKing’s College London 4616 LondonWC2R 2LSU.K.; 2 Department of Computer ScienceJohns Hopkins University 1466 BaltimoreMD21218USA; 3 ICube institute, CNRS, IHU Strasbourg, University of Strasbourg 27083 67081StrasbourgFrance; 4 Fakultät für InformatikTechnische Universität München 9184 80333MunichGermany

**Keywords:** Artificial intelligence, computer-assisted interventions, context-aware user interface, data fusion, interventional workflow, intraoperative imaging, machine and deep learning, surgical data science, surgical planning, surgical scene understanding

## Abstract

Data-driven computational approaches have evolved to enable extraction of information from medical images with reliability, accuracy, and speed, which is already transforming their interpretation and exploitation in clinical practice. While similar benefits are longed for in the field of interventional imaging, this ambition is challenged by a much higher heterogeneity. Clinical workflows within interventional suites and operating theaters are extremely complex and typically rely on poorly integrated intraoperative devices, sensors, and support infrastructures. Taking stock of some of the most exciting developments in machine learning and artificial intelligence for computer-assisted interventions, we highlight the crucial need to take the context and human factors into account in order to address these challenges. Contextual artificial intelligence for computer-assisted intervention (CAI4CAI) arises as an emerging opportunity feeding into the broader field of surgical data science. Central challenges being addressed in CAI4CAI include how to integrate the ensemble of prior knowledge and instantaneous sensory information from experts, sensors, and actuators; how to create and communicate a faithful and actionable shared representation of the surgery among a mixed human–AI actor team; and how to design interventional systems and associated cognitive shared control schemes for online uncertainty-aware collaborative decision-making ultimately producing more precise and reliable interventions.

## I. Introduction

Contemporary progresses in machine learning and artificial intelligence have permitted the development of tools that can assist clinicians in exploiting and quantifying clinical data including images, textual reports, and genetic information. State-of-the-art algorithms are becoming mature enough to provide automated analysis when applied to well-controlled clinical studies and trials [1], [2], but adapting these tools for patient-specific management remains an active research area, with the bulk of the research community having focused on fully automated machine learning tools. These considerations become especially critical in the highly heterogeneous context of surgery and interventional procedures that require patient- and team-specific decision support tools being able to draw information from nonstandardized interventional devices integrated into diverse interventional suites. Compared to computational tasks in radiology, the domain of computer-assisted intervention further creates unique methodological challenges, such as imposing stringent time constraints in the interventional suite, requiring knowledge of procedural data, and needing methods that deal with dynamic environments.

In this article, keeping a focus on imaging data, we review existing work and share insights on future developments of machine learning strategies that decipher, support, augment, and integrate into various surgical and interventional workflows while providing the flexibility required by clinical management. Flexibility is, for example, mandated to be able to deal with missing input sources, react to real-time user feedback, adapt to the patient risk aversion and preferences, handle uncertain or contradictory information, learn from potentially small and heterogeneous data, and so on. All of them are common in computer-assisted interventions. Imaging sources of particular interest for surgery and intervention include a wide range of well-known interventional modalities, such as surgical microscopy, video endoscopy, X-ray fluoroscopy, and ultrasound, more emerging biophotonics imaging modalities, such as hyperspectral imaging, endomicroscopy, and photoacoustic imaging, and also span classical radiology modalities, such as MRI and CT, that remain the main sources of imaging data for preoperative intervention planning and postoperative assessment. We argue that the stringent need to consider the context when analyzing surgical and interventional data coupled with the heterogeneity of information sources and domain knowledge in computer-assisted intervention applications calls for the development of novel domain-specific contextual artificial intelligence solutions, a domain that we coin as the contextual artificial intelligence for computer-assisted intervention (CAI4CAI). Feeding into the broader field of surgical data science [3]–[5], CAI4CAI will focus on the underpinning machine learning methodology exploiting contextual information and human interaction to enable the required responsiveness to deliver the clinical impact on surgery and interventional sciences.

To support our claim, we highlight some of the transformative machine learning research results and methodologies currently being developed across the spectrum of tasks in computer-assisted interventions. The impact of machine learning in intervention planning is discussed in Section II, intraoperative data fusion in Section III, intelligent intraoperative imaging in Section IV, surgical and endoscopic vision in Section V, and clinical workflow monitoring and support in Section VI. In these sections, we will highlight how flexible deep learning-based tools are becoming critical for the design of effective and efficient intervention planning solutions. During surgery, navigation solutions are often used to map preoperative information in the context of the intervention. However, navigation does not account for intraoperative changes. Learning how to coregister images is now leading to intraoperative registration solutions that are able to cope with the highly challenging task of aligning preoperative to intraoperative images coming from different imaging modalities. Concurrently, AI methodology is advancing to go beyond traditional navigation-based data fusion and image overlay to exploit information coming from complex or synergistic data sources. This is giving rise to what we refer to as intelligent intraoperative imaging. Data-driven modeling strategies coming from the computer vision community are acting as instrumental starting points to achieve semantic information extraction from interventional data sources, including endoscopic videos, with applications ranging from automated polyp detection to surgical activity recognition. To deliver improved clinical outcomes through AI, all these building blocks are increasingly being integrated at the level of the complete surgical workflow with applications spanning the full breadth of surgical data science. In this area, starting from the data-driven mapping of clinical workflow and skills assessment, AI is now helping make contextual decision support tools and conditionally autonomous intervention a reality. Finally, closing thoughts are provided and further budding applications of CAI4CAI are discussed in Section VII.

## II. Intervention Planning

### A. Clinical Adoption of Intervention Planning Tools

Once a decision is made for a patient to undergo an interventional procedure, for any nontrivial operation, patient-specific planning of the intervention is required. The steps involved usually necessitate the acquisition of reference preoperative imaging data, semantic segmentation of anatomical structures in these images, determination of the surgical approach, and elaboration of an intraoperative plan leading to optimal outcomes for the patient. Such a plan might encompass establishing a surgical path and target, designing, or selecting a patient-specific implant or assistive adjunct tool such as a drill or saw guide [6]. In the majority of cases, such intervention planning is performed by a team of healthcare professionals, each with their own expertise, known as the multidisciplinary team (MDT). Relatively, little computer assistance is currently available for interventional planning in the clinic. Notable exceptions can be found in the field of neurosurgery, oral and maxillofacial surgery, and orthopedic surgery. What these specialties share is a relatively static surgical scene due to the proximity of rigid bone structures. Computed tomography (CT) provides a rich source of 3-D imaging information in this context. Indeed, due to the quantitative nature of CT images and the good contrast of bone, automated segmentation of bone has proved to be clinically reliable. Because of the seminal work of the Retrospective Registration Evaluation Project (RREP) [7], it is also clear that preoperative rigid registration of different imaging modalities, such as MR and CT, provides a robust means of fusing soft tissue contrast information with accurate bone delineation for neurosurgical planning. Such technical advances have supported the adoption of stereotactic surgery as a means of accurately targeting and guiding instrument toward deep-seated brain structures for procedures, such as brain biopsies for tumor grading and electrode implantation for the treatment of movement disorder or the localization of epileptic seizure onset zones. While computer-assisted surgical planning and subsequent surgical navigation become standard of care in neurosurgery and a few other disciplines, even in these fields, there is major scope to make the workflow more efficient through the development of further machine learning-enabled computer assistance.

### B. Machine Learning in Interventional Planning

Commercial surgical planning products are still limited in the automation they support, with many of the most advanced ones essentially relying on classical image analysis methods, such as atlas-based segmentation [9], to delineate soft-tissue structures of interests for a patient showing no gross pathological brain changes. Clinicians are often left with manual or generic interactive methods to delineate other structures of interest and define their surgical plan. When interventional planning only relies on the clinician getting a volumetric representation of the patient anatomy from preoperative data, advanced visualization techniques, such as cinematic rendering [10], can be considered as alternatives to explicit segmentation of structures. These may produce results that are less sensitive to noise and data variability but do not enable more quantitative planning. Developments of deep machine learning segmentation algorithms dedicated to medical imaging [11], [12] are rapidly changing to a level of accuracy at which automated segmentation of structures of interest can be done in a population of patients even in the presence of gross pathological changes [13]. However, many challenges remain for these tools to become of practical use for intervention planning purposes. Poor generalization, when faced with slight domain changes, is a recognized problem in the entire medical imaging community including on the diagnostic side. Expanding the size of the data sets on which deep learning algorithms are trained would certainly mitigate generalization issues by providing a much larger variety of training cases. Collaborative efforts within the community are notably focusing on providing open-access large annotated data sets for machine learning training purposes in some specific use cases [1]. However, collecting task-specific large annotated databases for medical imaging purposes faces its own challenges, given the time and expertise required to provide detailed annotations as well as the legal, privacy, and storage questions pertaining to sharing large patient data sets across multiple sites. Federated learning for multi-institutional collaboration in medical imaging [14], [15] provides a potential technical solution to this problem. Implementing such solutions at scale will require concerted efforts reaching far beyond the methodological research community. Furthermore, changes such as device upgrades or challenges posed by new clinical indications will not be captured by increasing the pool of retrospective training data. Active research to address such inevitable but unpredictable domain gaps is rooted in domain adaptation techniques [16]. These advances are necessary for automated machine learning tools to make an impact on the clinical setting. Prospective randomized clinical trials (RCTs) are widely seen as the only source of trustworthy clinical evidence, yet studies implementing RCTs with systems relying on deep learning tools for medical imaging currently remain noteworthy exceptions [17].

### C. Importance of Flexible Contextual Machine Learning

**Fig. 1.**
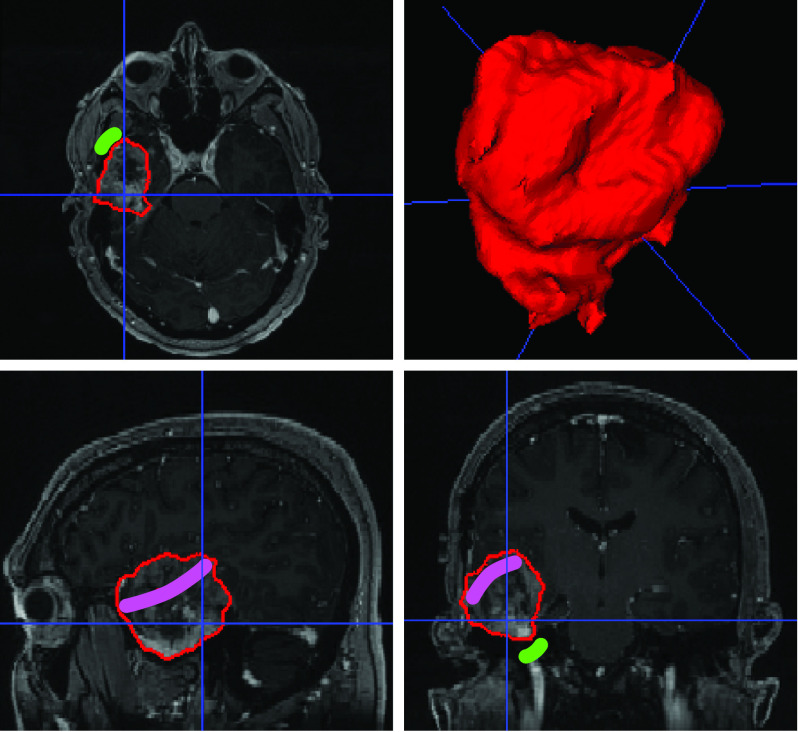
Interactive algorithms are required to deliver context-aware artificial intelligence. In this example, using the algorithm presented in [8], brain tumor segmentation is initially performed automatically using a pretrained algorithm. As a part of the surgical planning, the user may want to refine the segmentation by providing scribbles to denote areas that should be excluded (green region) or included (pink region) irrespective of the initial segmentation. The algorithm then adapts its output to respect the user input.

What distinguishes segmentation in surgical planning from segmentation in diagnostic imaging is, nonetheless, that the objective is not necessarily always that of reaching the best performance in getting the structures delineated with subvoxel accuracy. Surgical planning needs to respect the patient-specific needs and preferences of the surgeon. This requires putting the clinical team at the center and promoting flexible tools that integrate into the surgical workflow. Interactive deep learning methodologies are emerging to combine rich prior knowledge embedded in retrospective data from previous patients with as-sparse-as-possible annotations provided by clinicians [8], [18]. As illustrated in Fig. 1, deep interactive segmentation allows the clinical expert to refine the results from an initial automated step and, most importantly, to adapt the inferred results on the fly based on contextual information. Furthermore, given the heterogeneity and evolving nature of the surgical practice, additional flexibility is required to handle potentially missing input modalities. Recent work in deep machine learning is focusing on dealing with such dynamic heteromodal context while exploiting heterogeneous sources of data for the training process [19], [20]. Bringing flexible machine learning tools to maturity will certainly play an important role in supporting the clinical adaption of AI in surgery.

As highlighted earlier, segmentation of structures from preoperative images is often the foundation of computer-assisted surgical planning, and this currently remains the state of the art in many commercial solutions. Such static segmentation, when combined with intraoperative registration already, provides useful surgical navigation information for relatively static surgical scenes as is the case in neurosurgery. Nevertheless, computer assistance for intervention planning has the potential to provide impact much beyond the ability to automate the creation of 3-D anatomical models and overlay of functional data. Patient-specific simulation of given surgical plans has, for example, been introduced in orthopedic surgery with a long history in acetabular fracture surgery [21]. State-of-the-art orthopedic surgery planning systems allow to design patient-specific implants and patient-specific surgical guides by enabling the simulation of the effect of different implants and implantation strategy on key outcome-related parameters, such as the range of motion of articulation or the limb length [22]. However, these tools often ignore the effect of soft tissue in the simulation process and still require very labor-intensive work for the surgical team to design patient-specific plans. Expert systems capable of automatically optimizing the surgical plan for a given orthopedic surgery are now being developed [23] and promise to make surgical planning more efficient [24]. In the context of deep brain insertion of instruments, machine learning approaches capable of automatically planning trajectories of multiple instruments, to maximize the efficacy of the surgery while minimizing intraoperative risks and avoiding collisions between instruments, have demonstrated a significant reduction in planning time for the implantation of stereoelectroencephalography electrodes for epilepsy treatment [25] and for laser interstitial thermal therapy [26]. Contextual and flexible machine learning for surgical planning promises to push the boundaries of interventional planning by exploiting data-driven approaches and real-time user feedback to efficiently plan for complex situations. An instrument bending model was, for example, trained in [27] to predict the deviation between an original surgical plan assuming rigid electrodes and the actual electrode paths as measured on a postoperative CT. Provided reliable uncertainty estimates on the prediction can be achieved, embedding such deflection models in the trajectory planning is expected to improve the safety and accuracy of stereoelectroencephalography electrode implantation planning.

Effectively, planning is moving away from the extraction of information captured in existing data and representative of a given (preoperative) time point. Context-aware learning methods are now being developed to also predict therapy-related changes and better inform interventional planning. By exploiting computationally complex noninvasive cardiac electrophysiology modeling coupled with transfer learning approaches, Giffard-Roisin *et al.* [28] notably achieved online personalized predictions of electrophysiology cardiac resynchronization therapy responses, thereby paving the way for better patient selection and patient-specific therapy optimization. In nonquasi-static environments, surgical planning is currently further limited by our capabilities to predict intraoperative anatomical changes. In abdominal surgery, for example, segmentation of structures from preoperative images may inform the clinician about the relative spatial organization of lesions and vascular structures. However, at the onset of a minimally invasive procedure, gas insufflation is typically performed to create the surgical workspace. This has a serious impact on the geometry of the anatomy and challenges any attempt of intraoperative use of a 3-D model of the anatomy generated from preinsufflation images. Current approaches typically rely on focusing on smaller regions where rigidity assumptions between preoperative and intraoperative data may still hold [29], thereby limiting the scope of surgical planning. Data-driven prediction of anatomical changes relating to gas insufflation in laparoscopic surgery was proposed in [30]. Still, in the context of liver surgery, a system able to take into account nonimaging patient data and factual knowledge gathered from quotable sources, such as clinical guidelines, was proposed to support individualized treatment planning [31]. While relying on handcrafted features and exploiting models with limited expressiveness, this article paved the way for more holistic interventional planning. It is expected that the context-aware interventional planning will be informed by refined prediction models to suggest therapeutic plans cognizant of clinical experience as well as potential intraoperative changes and associated risks but also flexible enough to take into account any further input from the interventional team interacting with a responsive planning system.

## III. Intra-Operative Data Fusion

### A. Navigation and Image Registration Challenges

No matter how refined and capable interventional planning becomes, its full value for procedural guidance and intraoperative decision-making support remains contingent on appropriate geometric alignment with intraoperatively acquired data. This alignment is achieved using registration methods that either rely on dedicated external hardware, such as optical or electromagnetic tracking systems [32], or operate directly on intraoperative images [33].

Image-based registration in the interventional context has received substantial academic attention [34], [35]. This is because external navigation, while improving surgical accuracy, is associated with increased procedural time and complex and manual intraoperative calibration procedures that may lead to a high level of surgeon frustration [36]. It is widely believed that image-based registration will better integrate with procedural workflow, mitigating many negative aspects of external tracking approaches while providing similar accuracy. Furthermore, since no additional hardware is required, there is great potential for widespread adoption and deployment of these purely software-driven methods. This suggests that navigated surgery may also become available in remote and rural hospitals that could not afford dedicated equipment otherwise.

Despite the clear opportunity, image-based registration is not yet widely used in interventional clinical practice. This is because, depending on the clinical context, several challenges of image-based registration have not yet been solved reliably. During surgery, the anatomy undergoes highly complex deformations, including the loss of mass or topological changes during resections. Accurately recovering bio-mechanically plausible transformations that represent an anatomical change from preoperative to intraoperative state that is measured with different imaging modalities is the subject of the ongoing research. Here, we will focus on two of the associated challenges: 1) modeling image similarity between the images of the same anatomy but acquired with different modalities and 2) estimating initial transformation parameters that are good enough for registration algorithms to succeed.

On a high level, image registration seeks to find a transformation that, when applied to the moving image, aligns it with the target image such that the locations in both images are in correspondence. Quantifying *correspondence* is achieved using image similarity metrics that, usually, operate on the image intensity values. A straightforward comparison of intensity values, e.g., using a simple sum of squared differences, is generally unrewarding since the underlying assumption on image formation is prohibitively strong, even when moving and target images are acquired with the same imaging modality. For interventional image fusion, the problem is more challenging since images of different modalities must be aligned. In this case, the additive Gaussian noise assumption underpinning the sum of squared differences is certainly violated. Even worse, due to the different physical processes that govern image formation, there is no guarantee that the same anatomical structures are visible in both images, thereby challenging the adequacy of co-occurrence-based similarity metrics, including correlation and mutual information. Nonetheless, despite these limitations, model-based image similarity criteria currently remain the state-of-the-art performers in many interventional image-registration tasks, including ultrasound to MRI registration for neurosurgical guidance [37], [38].

### B. Contextual Learning for Image Registration

Using deep learning to go past some of the limitations of classical image registration is an active area of research. However, due to the fundamental challenge of gathering ground-truth data for image registration, many of the most successful learning-based registration methods for diagnostic images exploit unsupervised learning and optimize a classical image similarity metric-based loss [39], [40]. This approach remains unsuitable for most interventional purposes where more flexible solutions are required. A prominent example highlighting the need to take the interventional context into account is a transrectal ultrasound (TRUS)-guided prostate biopsy. Conventionally, the biopsy target is segmented on preoperative 3-D MR images, and this must then be registered to intraoperative 3-D TRUS volumes. Since MR and TRUS images exhibit a substantially different image appearance, contrast, and artifact level, this suggests that no good mathematical model exists to describe image similarity between these two modalities. Data-driven approaches that do not explicitly model intensity correlations to test for image correspondence but optimize a surrogate measure thereof now achieve state-of-the-art performance. One candidate surrogate measure can be defined by enforcing segmentations of the same structures to exhibit maximal overlap after registration [41]. Remarkably, learning to optimize for such losses does not require access to ground truth for the spatial transformation and leverages application-specific annotations that are considered as weak annotations. Further contextual information can be captured by learning data-driven spatial transformation models or regularization terms [42]. Related physics-based deformation models have been trained to predict shape changes in segmented organs from sparse annotations, which could be used for augmented reality purposes [43], [44]. Taking account of the interventional context one step further, Hu *et al.* [45] noticed that in many cases, including MR-TRUS-guided biopsy, the main purpose of interventional data fusion is to propagate a patient-specific target defined on a preoperative image to its interventional counterpart and proposed to replace the registration step by a conditional segmentation one.

Even in scenarios where data-driven similarity metrics may be learned, finding the transformation that optimally aligns a pair of images can remain nontrivial. This is because image similarity is well defined, i.e., informative, only in a narrowly circumscribed vicinity around the true transformation, emphasizing the need for appropriate initialization, such that the initial mismatch falls within the *capture range* of the image similarity metric and optimization algorithm [46]. While adequate initialization is challenging in all registration scenarios, it is considered to be most detrimental in slice-to-volume applications. Such applications are common in image-guided interventions, with the most prominent examples being the bijective alignment of 2-D B-mode ultrasound to 3-D MR or CT volumes or the projective registration of preoperative 3-D MR or CT volumes, or CAD models to intraoperative 2-D X-ray or endoscopy images.

In cases where the 3-D imaging protocol context is well defined, i.e., one is guaranteed to observe the same extent of anatomy, direct approaches to initialization are possible. These methods only accept the 2-D image as input and directly estimate its initial pose relative to a 3-D canonical atlas coordinate system that is implicitly defined by the choice of 3-D image database [47], [48] or tool model [49]. These approaches are attractive, mainly due to two reasons. First, run times are short since only 2-D images must be processed. Second, they lend themselves well for scenarios where 2-D slices are acquired successively to reconstruct a full 3-D volume. However, due to the complexity of the problem and canonical atlas assumption, their performance is often limited in practice.

When a canonical space cannot be defined, alternative approaches typically mimic the external tracking workflow where relative poses are inferred analytically. While external tracking devices require attachment or implantation of artificial fiducial markers to get position information readouts, AI-based approaches seek to establish correspondence directly from the images or from sparse but corresponding image locations. In [50], by learning from a data set of tracked ultrasound, the authors demonstrated that without inference-time reliance on the tracker, deep learning approaches can estimate the 3-D motion occurring in between consecutive 2-D ultrasound images with an accuracy far exceeding that of conventional speckle decorrelation techniques and matching that of the external tracker. This allows for a sensorless 3-D freehand ultrasound and creates new opportunities in computer-assisted interventions. Another complementary powerful concept for trackerless image alignment is the detection and identification of anatomical landmarks. These are particularly appealing since they carry semantic meaning and, consequently, define point correspondence across modalities and domains. Reliably detecting anatomical landmarks is complicated because of changing appearance based on viewpoints but has recently become possible due to powerful convolutional neural network-based image analysis for anatomical landmarks, as shown in the pelvis [46], [51] and knees [52]. The same concept of point correspondence naturally extends to tools and implants where, rather than relying on anatomical landmarks, keypoints on the CAD model are used [53]–[55]. The aforementioned approaches aim at discovering the well-defined points; however, finding the same arbitrary point in multiple images is equally appropriate to establish correspondence. In this formulation of the problem, an AI-based algorithm is trained to produce a pose invariant latent representation of point appearance. Then, query points can be randomly sampled in one image that is then rediscovered in the target image [56], thereby establishing correspondence. This approach is appealing since it does not impose any prior on the imaged object; however, learning a pose invariant latent representation so far has only been demonstrated for comparably small pose differences.

## IV. Intelligent Intra-Operative Imaging

### A. From Data Fusion to Intelligent Imaging

Intelligent intraoperative imaging refers to augmenting the value of intraoperative images for clinical decision-making by providing additional information that is tailored to the context of the intervention. In increasingly granular order, the context here describes the interventional requirements specific to a certain procedure, step in the surgical workflow, decision, or even surgeon’s preferences. So far, efforts in this direction are dominated by data fusion methods that seek to enrich intraoperative images with procedural planning information that exists from preoperative data. While this approach, even when relying on classical CAI tools, has been deployed successfully for several types of procedures [33], it is fundamentally limited in its capabilities of fully leveraging all acquired data. This is because the value of intraoperative images is reduced to a proxy to support, e.g., image-based registration or as a means for overlay, while all *intelligent information* that really augments the decision-making is propagated solely from preoperative images. In addition to underexploiting intraoperative images, this strategy only allows for displaying information derived from preoperative data that become outdated as surgery progresses. This calls for the development of intelligent intraoperative imaging that fully leverages the information contained in interventionally acquired data in real-time. Augmenting decision-making in this way offers clear opportunities by: 1) automating quantitative measurements required for precision medicine and 2) extracting information that is otherwise not easily accessible, which may allow the development of new surgical techniques. Still, contextual and intelligent interventional image analysis is not yet the mainstream technology because, compared to diagnostic image analysis, the environment for developing AI solutions is even more hostile. From our experience working with clinical collaborators across different sites and specialties, we believe that this is primarily due to three reasons. First, while hundreds of images are acquired for procedural guidance, only very few, if any, are archived [58]–[60], thereby suggesting a severe lack of meaningful data for researchers to work with. Second, learning targets beyond segmentation are not well established or defined. Third, images of the anatomy are acquired from multiple viewpoints, the exact poses of them are not reproduced nor known. Finally, the overall variability in the data is further amplified by surgical modification of anatomy and the presence of tools. Overall, the accessible data are heavily unstructured and exhibits enormous variation, which challenges meaningful data augmentation strategies. As a consequence, in order to train AI algorithms on interventional images, solutions to the data set curation and annotation problem must be found first. Overcoming these hurdles seems challenging and is reflected in the observation that only very little work has considered learning in this context. It is worth mentioning that the lack of annotated and/or paired data equally affects other methods presented in this article.

### B. Simulation-Based Training

**Fig. 2.**
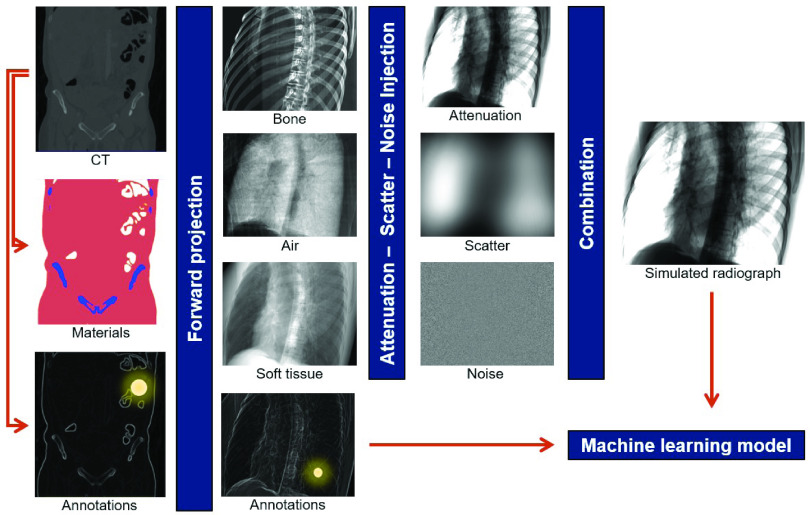
Realistic simulation of X-ray image formation from preoperative CT is one possibility to create large quantities of well-annotated images. Pipeline represents the simulation approach described in [57].

Initial steps in addressing the data problem have been taken, serving as a stepping stone for the transformative technology that is *intelligent imaging*. While the large-scale acquisition of highly structured data is tractable for some interventional applications, particularly ultrasound [61], [62], most other approaches rely on synthetic data generation from physical models of the scene. This paradigm is attractive because all quantities of interest are precisely known by design; however, if the simulation is performed naïvely, AI models trained on synthetic data will not generalize to clinically acquired images because of the large domain mismatch paired with poor generalizability of today’s models [57]. Three complementary ways have recently been shown to mitigate this problem. First, if the clinically acquired data are available in addition to the well-annotated synthetic data, style transfer algorithms can be trained that alter the appearance of real data to close the domain gap, as shown for the ophthalmic surgical microscopy [63], [64]. Using such enhanced simulated data for training of more complex tasks has been applied successfully to endoscopy [65] and X-ray imaging [66]. Second, if too little clinical data are available, learning a style transfer algorithm is impossible. In these cases, a powerful alternative is increasing the realism of synthetically generated images in a model-based approach. Doing so requires accurate models of all physical principles that govern image formation; however, approximations are usually required to reduce simulation time to acceptable levels. Realistic simulation works well for X-ray-based modalities, as illustrated in Fig. 2 and demonstrated in [57] and [67]. It has also been proposed in endoscopic imaging [68]. However, the level of required realism likely depends on the application and learning target since it has been shown that even less realistic simulations could be adequate, e.g., in some ultrasound applications [69]. The aforementioned approaches aim at reproducing the real data appearance that is very complicated in practice. If closely matching real data appearance is found to be impossible, domain randomization can be used to improve the robustness of the trained model to partially unseen data. Rather than perfectly matching real data characteristics, the goal of domain randomization is to generate multiple versions of the same sample with all but the important characteristics randomized. When training AI algorithms on such data sets, the models are assumed to become robust to these types of domain changes. Domain randomization can be seen as image formation-based data augmentation and has recently been applied to X-ray imaging [70] as well as colonoscopy [68], where achieving realistic image appearance is very complicated due to fine texture and specular reflectance of the tissue. It is worth mentioning that all the above-mentioned techniques for synthetic data usage are similar in that AI algorithms never process real data during training. This characteristic is associated with a notable drop in performance when applied to real data due to residual domain mismatch. Consequently, assessing algorithmic performance only on a synthetic test set will severely overestimate the AI models accuracy during deployment and quantitative experiments on clinical data are required. Ultimately, training the AI directly on real data is preferable, highlighting the need for further research on unsupervised and self-supervised learning to leverage large quantities of unlabeled data.

### C. Intelligent Imaging in Interventional Biophotonics

Although conventional interventional imaging, such as X-ray fluoroscopy, surgical microscopy, endoscopy, and ultrasound, will benefit from being augmented by contextual AI, another interesting area in which the intelligent imaging paradigm is expected to make an important impact is that of the interventional biophotonics imaging. The initial focus in biophotonics has been on developing optimal, task-specific, contrast agents that would be merely be directly visualized, e.g., in tumor-specific fluorescence imaging. The biophotonics community has, however, faced stringent challenges in identifying versatile contrast agents suitable for use in patients and realized that tissue differentiation would remain challenging with such an approach. Advanced high-dimensional optical imaging techniques are currently seen as promising solutions for intraoperative tissue characterization, with the advantages of being noncontact, nonionizing, and noninvasive or minimally invasive. However, because of the high-dimensional nature of the generated data, direct visualization by the clinical team becomes impractical. This calls for automated learning-based information extraction before display. As in the previous examples of intelligent imaging, many of the most advanced AI-supported interventional biophotonics imaging devices currently exploit model-based learning or unsupervised learning. Point-based measurement devices able to measure the Raman scattering have recently been translated into commercial products [71] with support from supervised classification [72] or unsupervised dimensionality reduction [73]. Addressing the lack of wide-field information in point-based systems, the community has looked into modalities such as hyperspectral imaging [74] with an increasing use of machine learning to solve some of the intrinsic challenges of high-dimensional data. Indeed, while bearing rich information, the raw 2-D -space + wavelength + time data that hyperspectral imaging produce are difficult to interpret for clinicians as it generate a temporal flow of 3-D information that cannot be simply displayed in an intuitive fashion. Innovative use of invertible neural networks in combination with model-driven simulation has been used to train neural network-based regressors that are capable of real-time operation and can provide uncertainty estimates for oxygen saturation measurement from hyperspectral data [75]. Unsupervised deep manifold embedding for hyperspectral imaging was proposed in [76], and deep learning was used for reconstruction from sparse hyperspectral data [77]. Intelligent imaging concept with simulation- or model-based trainings are also being progressed with other emerging biophotonics imaging modalities, such as for superresolution in endomicroscopy [78], [79], and artifact suppression in photoacoustic imaging [80].

### D. Toward Prospectively Planned Intelligent Imaging

With the availability of training data, via either dedicated data collection or synthetic generation, AI algorithms can be developed to analyze intraoperative images in near real time and supply contextual information to improve decision-making. Omitting applications to endoscopic video sources that are discussed in depth in Section V and focusing first on the interventional X-ray imaging, benefits of real-time machine learning range from segmentation of tools [53], [81], [82], anatomical landmark detection [51], [52], anatomy localization [83], and denoising [84], [85], to surgical phase recognition [81]. Corresponding developments can be found for ultrasound imaging [86]–[88].

While the above-mentioned list of applications merely hints at the potential that AI-based analysis of interventional images has to offer, there is an interesting observation: the majority of *intelligent imaging* algorithms, including all the aforementioned methods, try to provide richer information by the automated analysis of traditionally acquired images, with little or no knowledge of the image acquisition workflow. This raises an interesting question: if it is known what information is desired or desirable at any given point during the surgery, is it possible to prospectively acquire an image that is most informative in that particular context? Initial steps in this direction have recently been reported, exploiting ultrasound image formation to suppress scatter [89] or beamforming a B-mode image [90], [91] together with producing its segmentation [69]. Zaech *et al.* [92] use an AI-based algorithm to recommend task-optimal and patient-specific C-arm X-ray trajectories during cone-beam CT of spinal fusion surgery, and similar ideas arise for ultrasound transducer positioning [93].

The domain of real-time interventional image analysis is fairly untapped as of yet but offers great opportunities for workflow analysis, surgical progress monitoring, including anticipation and adverse event detection, and supplying rich information for human-in-the-loop decision-making. In addition, task-aware and autonomous imaging modalities may benefit interventional imaging already one step before the image is analyzed and may, thus, give rise to disruptive technology and novel surgical approaches.

## V. Surgical and Endoscopic Vision

### A. Recognizing Endoscopic Activity

Standard endoscopic imaging is certainly the modality most closely relating to natural images. It should, therefore, not be surprising that machine learning tools for interventional images have developed most rapidly in this field. As a proxy for the eyes of the surgeon inside the patient, the endoscopic camera is the privileged source of digital information to understand the activities performed during endoscopic procedures. Endoscopic videos usually capture most of the activities performed within the patient. Recognizing and understanding these activities are essential to develop novel assistance systems that are reactive to the context, e.g., that can provide timely instructions to operating room (OR) staff, enforce safety checkpoints, or log automatically relevant information within the surgical report. Surgical activity recognition from endoscopic videos is, however, a highly challenging task due to the variability existing across patients, surgical treatments, and surgical teams.

In the recent years, a large body of work has focused on recognizing the surgical steps of a procedure directly from the videos [94]–[99]. This has notably been the case in cholecystectomy, a common procedure consisting in removing the gallbladder, which is frequently used in research due to its high frequency of occurrence and well-standardized protocol [100]. There, the steps include, for instance, “the Calot triangle dissection, cystic duct and artery clipping and cutting, gallbladder dissection, and gallbladder packaging.” Recognition of these steps allows for the automated understanding of the progress of the surgery. To perform recognition, models of the underlying workflow of the procedure are learned from data sets of exemplary videos, annotated manually with the different steps. In [97], the model consists, for example, of a visual feature extractor relying on a deep neural network that feeds a temporal recognition model, such as a hierarchical hidden Markov model or an LSTM model. Several types of procedures have been successfully studied for step recognition besides cholecystectomy. Examples are cataract surgery [95], [96] and laparoscopic sleeve gastrectomy [98]. As the current recognition methods show very promising results and real-time capabilities, they can potentially be directly embedded in the endoscopic tower to deliver contextual support. Other interesting prediction tasks have been tackled with success using deep learning methods. In [101] and [102], the remaining duration of the procedure is predicted in real time using deep recurrent models trained directly from video data. In [97], [103], and [104], the presence of the instruments in the surgical scene is automatically detected. Additional applications include bleeding and smoke detection [105], [106], as well as surgery type identification at the beginning of the procedure [107].

Beyond the recognition of the surgical steps indicating the progress of the surgery and the recognition of events, such as bleeding, many potential applications, such as safety monitoring and human–robot cooperation, require a finer level of understanding of the surgical activities. Future research, therefore, needs to demonstrate accurate recognition of the detailed interactions between the tools and the anatomy. To have an impact beyond a single OR, recognition methods will also need to scale up to different types of surgeries, ORs, and hospitals without requiring the manual annotations of large data sets for each situation. Recent methods exploiting nonannotated videos through self-supervision or weak-supervision [104], [108]–[111] or exploiting synthetically generated surgeries [64] may prove very useful to train the next generation of surgical recognition systems.

### B. Understanding Image Semantics

Understanding the surgical scene from the endoscopic images is fundamental for context-aware intelligent computer-aided assistance. During augmented reality visualization, precise pixel-based segmentation of the tools is necessary for handling occlusions and providing the user with the correct perception. Implementing safety warnings, such as no-go zones, requires the detection of critical anatomy. When another imaging modality is used, its registration to the endoscopic video may require the localization of anatomical landmarks [113]. Similarly, implementing degrees of autonomy during robotic surgery requires the localization and recognition of the neighboring tools and anatomy.

Recently, a large body of work has targeted the detection and segmentation of surgical instruments [114]. Deep learning methods have been proposed for both bounding box or articulated tool detection [115]–[117] and for pixel-based tool segmentation [118], [119]. Their superiority has been confirmed on laparoscopic and surgical microscopy data sets in two international challenges organized in 2015 and 2017 at the MICCAI conferences [120], [121]. Still, the data sets used for evaluation are limited in size and variability. They are far from representing the diversity of surgical scenes, which can indeed be very challenging due to the presence of occlusions, smoke, bleeding, specularity, motion blur, and deformation. Furthermore, the aforementioned approaches are fully supervised and, therefore, impose an important burden on the collection of representative training data sets. New approaches are needed that can generalize easily to various types of procedures and be trained using weaker information for training, such as image-level tool presence [104], point annotation [122], or scribbles [123].

Far less work has addressed the much needed anatomy detection and segmentation, certainly due to the lack of available public data sets. The community is, however, putting large efforts in this direction, as illustrated by the recent generation of the CaDIS data set [124], which contains pixel-level annotations for 36 semantic classes in cataract surgery videos. Progress has also been achieved in specific areas, such as liver segmentation [125], lesion detection and characterization during gastroscopy [126], or polyp detection during colonoscopy [17], [127]. Here, again, deep learning is the state of the art, as demonstrated for polyp detection in a challenge organized at MICCAI 2015 [128]. Due to the real-time capabilities of deep learning approaches, the intraoperative benefits of such systems already start to be evaluated in RCTs [17].

### C. Reconstructing Anatomic Geometry

**Fig. 3.**
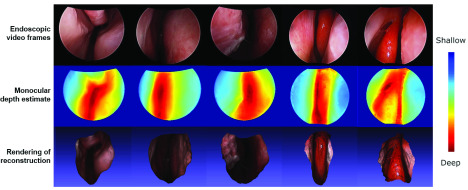
Endoscopic video (top), monocular depth estimate (middle), and rendering of a photorealistic reconstruction (bottom). Results were achieved using the self-supervised method described in [112].

Endoscopy mimics the surgeon’s eyes within the body, but due to the monocular construction of endoscopes, it lacks one important visual cue: depth. This shortcoming has implications: it has recently been shown that the availability of 3-D anatomic geometry benefits several clinical tasks, including the detection of critical anatomy, such as polyps [129], and the registration of preoperative 3-D data to endoscopy video to enable navigation [130]. In addition, analyzing 3-D representations of anatomy would allow for the introduction of quantitative measurements, enabling the standardization of clinical reporting across sites. Recovering anatomic 3-D geometry, e.g., to augment endoscopic video with depth cues or to provide dense 3-D reconstruction, has gained considerable traction and is now an emerging discipline with developments often orthogonal to those for complementary tasks, e.g., segmentation. This is because deep learning-based algorithms are able to exploit image-level features to provide dense depth estimates even from monocular video, complementing traditional optical endoscopy with depth sensing as “pseudomodality.” However, training depth estimation algorithms on endoscopic sequences is complicated in practice because no paired depth measurements exist naturally. While paired data can be generated in silico via simulation from CT [65], [68], [131], the resulting trained models will need to overcome the domain mismatch to real clinical data with methods similar to that presented in Section IV. Recently, self-supervised training paradigms that rely on traditional multiview stereo approaches have received increasing attention as they can be trained directly and solely from the endoscopic video. Multiview stereo algorithms, including structure from motion [112], [130] and simultaneous localization and mapping [132], can be adapted to work with endoscopic video, but they cannot provide dense 3-D reconstructions due to the lack of photometric constancy in endoscopic video and texture scarceness that complicate feature matching across frames. These algorithms do, however, provide a few reconstructed 3-D points and, more importantly, relative camera poses that can be used to supervise monocular depth estimation [112], [132]. A representative photorealistic reconstruction achieved using a structure from motion supervised depth estimation method is shown in Fig. 3. These methods achieve state-of-the-art performance with good generalization ability; however, the resulting reconstructions are only up to scale. Among the biggest premises of video-based reconstruction is the possibility of monitoring anatomical change during surgery. This would require methods to robustly handle various sorts of uncontrollable variation, including bleeding, smoke, or tool presence. Solutions to these problems are currently unknown. Even in more controlled scenarios, widespread adoption of learning-based reconstruction from the endoscopic video is hindered by the lack of publicly available data sets, making it unclear how well today’s algorithms perform on clinical data. This challenge is further aggravated by the lack of direct evaluation targets. When applied to real clinical data, current reconstruction or dense estimation algorithms can only be evaluated via surrogate tasks, such as video-CT registration [112], [133] or polyp classification [129].

## VI. Clinical Workflow Monitoring and Support

### A. Notion of Surgical Control Tower

While imaging alone provides valuable information, modern procedures rely increasingly on a variety of complex devices and intricate workflows. This limits the knowledge extraction that AI systems can do based on imaging alone and makes it difficult for humans to properly analyze in real time the wealth of available data. Furthermore, even though the quality of care has generally improved with the introduction of new surgical techniques and devices, adverse events still occur, a large part of that are preventable [135], [136]. Humans are prone to fatigue, teams to miscommunications, devices can fail, and for all roles, surgical tasks require an ever-increasing level of specialization. The increased use of digital equipment in the OR, however, opens up new opportunities for support and monitoring, at the level of the whole room, by providing artificial intelligence systems with real-time data that capture a faithful representation of the processes taking place during the surgery. Indeed, most of the activities happening in the room can be captured digitally either through interactions with equipment, such as information systems, room control interfaces, imaging devices and instruments, or through the use of sensors, such as ceiling-mounted cameras, which are now becoming widespread and increasingly used for documentation, teaching, and augmented reality assistance. Consequently, it is highly likely that in the near future, assistance systems will be fully integrated in a digital OR that will monitor surgical processes through AI, akin to a *surgical control tower* [137], [138], that can analyze the whole digital information in real time to provide context-aware support and information within and outside the OR. Applications for such a control tower are, for instance, the transmission of live information about the OR status, the adaptation of user-interfaces to the surrounding context, the display of instructions within the OR, the creation of an automated report, the recording of the activities for archiving and legal purposes, the enforcement of safety checklists, the detection of anomalies with respect to past workflows, and improved scheduling for staff and patients. To perform these tasks, the control tower will have access to and crunch masses of multimodal digital data coming from hundreds of past surgeries.

### B. Endeavor Rooted in Surgical Data Science

**Fig. 4.**
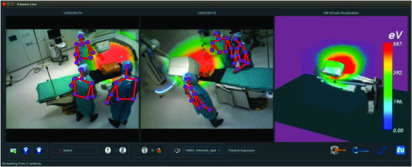
Capturing the 3-D context of the OR is necessary for providing AI-based decision support and monitoring risk. In this example, the staff radiation exposure during an X-ray-based procedure is computed *in situ* via simulation and displayed with augmented reality in a training scenario [134].

An essential component of the control tower is the data-driven modeling and understanding of the clinical activities, an undertaking that taps into the emerging research field of surgical data science [3], [4]. Machine learning has been key to generate models of procedural interventions from data [139], [140], and ontologies have also been developed to standardize the resulting models [141]. Implementations of such AI-based applications start to emerge in various institutions, besides the ones focusing on analyzing endoscopic videos already mentioned in Section V. As video data remain one of the main sources of information, they highly rely on deep learning. Videos captured by the cameras mounted in the room provide indeed a rich source of information about the activities without disrupting the workflow. For instance, a patient and staff radiation exposure monitoring system for hybrid procedures illustrated in Fig. 4 was proposed in [134]. It relies on several RGB-D cameras to estimate the 3-D pose of the persons and room layout, which can then be used to simulate and visualize *in situ* X-ray propagation around the patient table. Haque *et al.* [142] develop a system to monitor hand hygiene in hospital corridors in order to analyze and reduce the hospital-acquired infection. The approach uses a large set of depth cameras installed to observe the hand-soap dispensers. For the intensive care unit, Ma *et al.* [143] and Yeung *et al.* [144] present methods based on color or depth video data for the detection of patient mobilization activities. Key building blocks to the success of these applications are the estimation of clinician and staff poses [145]–[147], as well as the recognition of their activities [148]–[151]. As for traditional visual data, deep learning-based approaches are currently the best-performing methods for these tasks though it should be noted that they do not necessarily perform as well on clinical data yet. This is due to the specificity of clinical videos, where staffs wear gowns and masks, colors are often similar, and cameras observe the room from restricted positions, but also from the fact that there is no clinical COCO or Imagenet data set yet. Srivastav *et al.* [152] evaluate the state-of-the-art human pose estimation approaches, and Issenhuth *et al.* [153] evaluate the state-of-the-art face detection approaches on clinical data. Both studies show a large margin for improvement. Since the development of large annotated data sets of clinical videos may be difficult due to the expertise required and the restrictions on data, other approaches need to be developed, for instance, using the nonannotated data for transfer learning [153].

This will also help deploy the surgical control tower in new clinical environments, as the variability in room layout, camera configuration, and workflow can be high. Retraining the assistance systems using only nonannotated data from the novel environment or a tiny subset of annotated data will be crucial for the adoption of these technologies. As even the collection of nonannotated video data can be challenging due to data and privacy regulations, it may also be required to implement federated learning approaches or develop methods that are able to cope with privacy-preserving data, such as depth-only videos [142] or even low-resolution depth videos [154]. In [154], it is shown that 2-D human pose estimation can be achieved with reasonably high accuracy on depth images downsampled by ten to the resolution of $64\times48$. By using other information, such as system events [155] or speech analysis [156], the analysis of clinical activities will be further improved.

## VII. Discussion and Conclusion

While AI is starting to impact CAI, as described in this article, there is a number of challenges that are specific to surgery and intervention to overcome to deliver clinical impact. The leveraging context within learning paradigms will be crucial to address those in a clinically meaningful way. The emerging field of CAI4CAI offers researchers a large set of open problems to tackle. These notably stem from the heterogeneity of surgical procedures and their particular requirements for intraoperative imaging [157], the difficulties in data acquisition, the complexity in modeling and inferring decision-making processes, and the intricacy of the execution of surgical tasks. Over the years, the CAI community has defined increasingly powerful surgical process models [158] to gain an actionable understanding of surgical procedures while describing interventions as a sequence of tasks and activities at different granularity levels. At the finest level, mapping what should be the *Language of Surgery* [159], researchers currently break down surgical gestures into semantically relevant motion units called *surgemes* that are further composed of sequences of motion primitives named *dexemes* [160]–[162]. However, this taxonomy mostly focused on the surgical action and, in particular, on surgical tool manipulation and could, thus, rather be considered as mapping the *Language of Surgical Dexterity*. This is already a laudable achievement and led to scientists and engineers being able to, e.g., quantify the success of a training program for executing different surgical actions [163], [164]. As suggested by the study conducted by Birkmeyer *et al.* [165] for bariatric surgery, surgical skills can be highly correlated with the surgical outcome for certain procedures. AI systems have been shown capable of evaluating technical skills using data from either training scenarios [166] or real procedures [167]. However, by severely underutilizing the rich information contained in other data sources, the *Language of Surgical Dexterity* is still not capturing the most complex aspects of surgical decision-making. To address the need to capture, understand, and support all the cognitive interactions and processes taking place in the OR, the surgical data science community will need to drive the deployment of real-time multimodal data acquisition systems that will be used routinely. At the same time, it will foster the development of new standards and regulations aiming at increasing the interoperability of data, devices, and models. This will directly benefit CAI4CAI by simplifying the implementation and training of learning algorithms involving databases from multiple institutions while maintaining privacy, e.g., through federated learning. CAI4CAI in combination with surgical data science and surgical process modeling could, thus, aim at defining and understanding the ultimate *Language of Surgery* based on a large number of heterogeneous data sources used continuously by surgeons and interventional teams to guarantee the best outcomes for a given procedure. As the field blossoms, CAI4CAI researchers will address some of the most rewarding questions in computer-assisted intervention. Could CAI4CAI allow us to learn how decisions are made, or missed, throughout surgical procedures? Could CAI4CAI support such decision-makings? Instead of going through the traditional path of segmentation, registration, navigation, and visualization, could contextual machine learning allow us to optimize these steps for each given objective and allow for real-time computation and feedback based on a large amount of heterogeneous data, including preoperative and intraoperative imaging, patient characteristics, and surgeon preferences?

With more capable and flexible learning paradigms, synergistic collaboration is expected to happen between humans and AI-powered actors. The field is already seeing exciting attempts to bring the user and the user experience at the center of our research questions. For example, novel spatially aware visualization beyond traditional user interfaces is explored in [134] and [168]. The challenge of improving human situational awareness in operating with solutions beyond visualization is addressed in [169] with the use of context-specific soundtracks. Introduction of novel multimodal interaction paradigms and technologies within ORs will require extensive use of machine learning to optimize the user interfaces and to provide maximally relevant information and support while preventing inattentional blindness [170]. By developing systems that are able to learn from previous surgeries performed by experts how to provide context-aware support and instructions directly in the OR, in the manner of a virtual coach, as in [171], AI could have a strong impact on improving patient care. This is another aspect of CAI4CAI that needs particular focus from the scientific community and requires MDTs, including clinicians, user experience experts, and machine learning scientists, to work together and come up with intelligent end-to-end CAI solutions.

Finally, in this article, we did not have a particular focus on robotics. However, both surgical robotics and robotic imaging will play increasingly crucial roles in the years to come. Machine learning is demonstrating convincing results in real-time tool tracking [118], [172]–[174]. This, for example, enables automatic positioning of intraoperative OCT imaging planes within surgical microscopy for ophthalmic surgery [119], [175]. Integration of robotics within surgical suites would require them to act intelligently and synergistically with the human team and to be fully context-aware at all moments. The wish to have real-time multimodal imaging requires full intelligence and automation. It also requires direct communication and collaboration between surgical robots, imaging robots, surgeons, and surgical teams. CAI4CAI will have the challenge of enabling such ultimate intelligence, which requires many years of research and development in many disciplines while remembering a past experience with the first generation of context-aware computing [176]. Not only does CAI4CAI offer numerous exciting research directions but it also promises to revolutionize surgery and, therefore, the future of healthcare at a global scale.
